# The Use of Formalin

**Published:** 1899-03

**Authors:** H. Jerome Allen

**Affiliations:** Washington, D. C.


ARTICLE V.

### THE USE OF FORMALIN.

DR. H. JEROME ALLEN, WASHINGTON, D. C.,

The substance now known synonymously as formaldehyde, formic aldehyde, methyl aldehyde, or oxymethylene, is a gaseous body formed from methyl (wood) alcohol by oxidation.

Formalin (which is the trade name adopted by the Schering Chemical Works to designate their product) is a saturated watery solution of the gas formaldehyde, and occurs as a neutral, colorless volatile liquid of a pungent odor and sharp taste, miscible in every proportion with water and alcohol.

Paraform (polymerized formalin, paraformaldehyd, paraformic aldehyde or tri-oxymethylene) results from a simple evaporation or heating of formalin, and appears as a white, indistinctly crystalline powder, which is stable under ordinary conditions, and is made into tablets and sold in that form for disinfection.

Paraform is volatilized by heat, and is soluble in hot water, the solution possessing the characteristics of ordinary formalin.

The gaseous compound was first discovered by Von Hoffmann in 1867, who produced it by passing the vapor of methyl alcohol mixed with air over platinum powder heated to redness. Later it was, and is still produced by the action of silent electric charges on a mixture of hydrogen and carbon dioxide.

Its germicidal properties remained unknow until discovered by Loew in 1888. Since then a host of authorities have recognized and investigated its properties, and have, as a rule, pronounced it far superior to any general disinfectant now in use.

"Formaldehyde has the chemical property of uniting with sulphureted or nitrogenous products of decay, fermentation or decomposition forming true chemical compounds, which are odorless and sterile, and these compounds are in most cases actually antiseptics themselves.

"It is from this property of combining chemically with albuminous or nitrogenous bodies that formaldehyde derives its germicidal and bactericidal power; since bacteria and micro-organisms generally are not only albuminoid in character, but their food is mainly albuminoid, and when formaldehyde is present it combines with both the bacteria and their food, thus destroying them as well as the possibility of their existence." (Lilly).

In this fact lies the surpassing value of formaldehyde over such disinfectants as corrosive sublimate, carbolic acid, lysol, etc., for albuminous matter is at once coagulated by contact with these agents, and the resulting antisepsis is superficial, while the formnldehyde solution being possessed of a chemical affinity for albuminoids, thoroughly impregnates (and consequently sterilizes) all such substances with which it comes in contact.

Partly as a natural sequence to this property is developed its power of hardening and preserving animal tissue; converting soft tissues to a hard, leathery mass, dependent upon the strength of the solution and the time of action.

This effect is due, as before stated, to its penetrating action, whereby it readily unites with the albuminoid substance in the protoplasm of the cells, and checks all putrefactive changes permanently in dead tissue, and for the time being checks the growth of cells in living tissue.

Another valuable property of formaldehyde is its absolute lack of toxicity when in contact with living tissue in almost any strength. Every paraform, which contains one hundred per cent. of formaldehyde, is perfectly harmless if accidentally swallowed, inasmuch as its conversion into the gaseous state is so slow at the temperature of the body that it does not act at all injuriously upon the mucous membrane of the alimentary tract. The possession of these properties by formaldehyde should give it the first place in the list of dental antiseptics and disinfectants. Already its use in the profession as a general disinfectant has become quite general within the past two years.

It is the active principle of all the lately introduced mouth washes and antiseptic solutions, and their proven superior germicidal properties are due to the presence of this agent.

Its remarkable property of existing in three available forms—gas, liquid, and solid—adapt it readily to all purposes and classes of disinfection, and it bids fair to become the universal disinfectant of the near future. A few of its dental applications may be enumerated as follows:

For occasional use in the office to destroy all noxious odors, disinfect the draperies and furniture, and purify the atmosphere, nothing is better than a Schering formalin lamp. which disinfects the premises by the production of formaldehyde gas by the thermal evaporization of paraform. The ease with which the disinfection can be accomplished is one of its principal recommendations.

In the evening before leaving the office, place from ten to twenty paraform pastils (according to size of room) in the receiver of the lamp, light it, close the premises tightly, and let them remain so until the next morning, when by opening the doors and windows all odor of the disinfectant rapidly disappears, and the office and its exposed contents have undergone a complete sterilization.

In the treatment of putrescent root-canals is formaldehyde particularly valuable. A twenty per cent. solution should be worked into the root until all fetid odor is destroyed. The contents should be thoroughly removed, and the root can be filled immediately by incorporating a minute quantity of paraform with the powder of the cement used in the filling.

Another method of treating a putrescent canal with either a blind or fistulous abscess at the apex is to clean out the contents thoroughly with a twenty per cent. solution, and then seal in a small quantity of paraform. The paraform is vapor ized so slowly at the temperature of the body that the canal and the apical tissues are kept in a continued aseptic condition, with the resulting death of the micrococci and their spores.

In personal practice two applications at intervals of a week have sufficed to give most encouraging and apparently lasting results. Several chronic abscesses of years' standing have healed up with surprising rapidity under this treatment. —*Dental Cosmos.*

